# Identifying microRNAs associated with tumor immunotherapy response using an interpretable machine learning model

**DOI:** 10.1038/s41598-024-56843-3

**Published:** 2024-03-14

**Authors:** Dong-Yeon Nam, Je-Keun Rhee

**Affiliations:** Department of Bioinformatics & Life Science, Soongsil University, Seoul, Republic of Korea

**Keywords:** Cancer genomics, Cancer microenvironment, Bioinformatics, Cancer genomics, Machine learning

## Abstract

Predicting clinical responses to tumor immunotherapy is essential to reduce side effects and the potential for sustained clinical responses. Nevertheless, preselecting patients who are likely to respond to such treatments remains highly challenging. Here, we explored the potential of microRNAs (miRNAs) as predictors of immune checkpoint blockade responses using a machine learning approach. First, we constructed random forest models to predict the response to tumor ICB therapy using miRNA expression profiles across 19 cancer types. The contribution of individual miRNAs to each prediction process was determined by employing SHapley Additive exPlanations (SHAP) for model interpretation. Remarkably, the predictive performance achieved by using a small number of miRNAs with high feature importance was similar to that achieved by using the entire miRNA set. Additionally, the genes targeted by these miRNAs were closely associated with tumor- and immune-related pathways. In conclusion, this study demonstrates the potential of miRNA expression data for assessing tumor immunotherapy responses. Furthermore, we confirmed the potential of informative miRNAs as biomarkers for the prediction of immunotherapy response, which will advance our understanding of tumor immunotherapy mechanisms.

## Introduction

Immunotherapy targets immune checkpoints and is a type of cancer treatment that modulates the immune system to eliminate tumor cells^1^. Immune checkpoint molecules, such as cytotoxic T-lymphocyte-associated protein 4 (CTLA4), programmed death-ligand 1 (PD-L1), and programmed death 1 (PD1), have been thoroughly explored and have emerged as targets in immune checkpoint blockade (ICB) therapy^2^. In comparison to conventional cancer treatment methods such as radiotherapy and chemotherapy, immunotherapy offers the advantage of patient-specific production and the potential for a sustained response with fewer side effects. However, the high cost of cancer immunotherapy and the fact that not all patients respond to ICB therapy have created significant barriers to treatment^3^. Therefore, predicting ICB response before treatment is crucial.

To address this problem, several methods have been developed based on computational analyses. For example, Litchfield et al*.* identified predictors of ICB responses through a meta-analysis of tumor mechanisms using whole-exome and transcriptomic data for checkpoint inhibitor (CPI)-treated patients and devised a machine learning approach (XGBoost) to predict ICB responses^4^. Moreover, Chowell et al*.* developed a machine learning model that predicted ICB responses by integrating genomic, demographic, and clinical data from patient cohorts treated with ICB across various cancer types^5^. Kong et al*.* proposed a network-based machine learning framework to predict the treatment response to immune CPIs^6^. Moreover, Zhang et al*.* devised a computational model, Tres, which leverages single-cell transcriptomic data to discern robust T-cell signatures associated with immunosuppressive signals. Tres has shown efficacy in predicting clinical responses to immunotherapy in melanoma, lung cancer, triple-negative breast cancer, and B-cell malignancies^7^.

A prominent computational method called Tumor Immune Dysfunction Exclusion (TIDE)^8^ has also emerged. This method is based on the premise that transcriptome signatures, among various factors (such as PD-L1 expression level^9^, neoantigen load^10^, immune infiltration level^10^, and tumor aneuploidy^11^) can serve as biomarkers that affect the effectiveness of ICB. To predict the response to ICB, the authors of TIDE focused on how tumors evade the immune system and categorized the tumor immune evasion mechanism into dysfunction and exclusion^12,13^. Dysfunction refers to the infiltration of cytotoxic T cells into the tumor at a high level. However, these T cells are in a dysfunctional state and are unable to attack the tumor cells. In contrast, exclusion involves immunosuppressive factors in the tumor that prevent T cells from entering. TIDE utilizes key gene signatures as biomarkers and measures the TIDE score to predict ICB responses, thereby offering insights into the two evasion mechanisms.

Recently, various studies have supported the role of microRNAs (miRNAs) in regulating immune responses with diverse effects on the immune system. miRNAs are non-coding RNAs approximately 22 nucleotides in length that participate in post-transcriptional gene regulation by forming pairs with mRNA; they are intricately linked to various diseases^14,15^. The expression patterns of miRNAs are disease-specific, including those in cancer, making them valuable for reflecting disease occurrence and differentiation states^16^. Consequently, understanding the function of miRNAs can aid in identifying the pathological mechanisms underlying diseases and can potentially be associated with the ICB response. For instance, miR-200^17^ and miR-424^18^ influence the interaction between the PD-1 receptor and PD-L1 ligand by modulating PD-1 expression, which is an immune checkpoint molecule. Furthermore, miR-138 binds to the 3'-UTR of CTLA-4 and PD-1, reducing their expression and serving as a tumor-suppressive factor. It has also been investigated as a potential component of novel immune treatments^19^. Given that miRNAs regulate gene expression and are closely associated with the immune system, they can be used as biomarkers to predict ICB responses.

**Figure 1:**
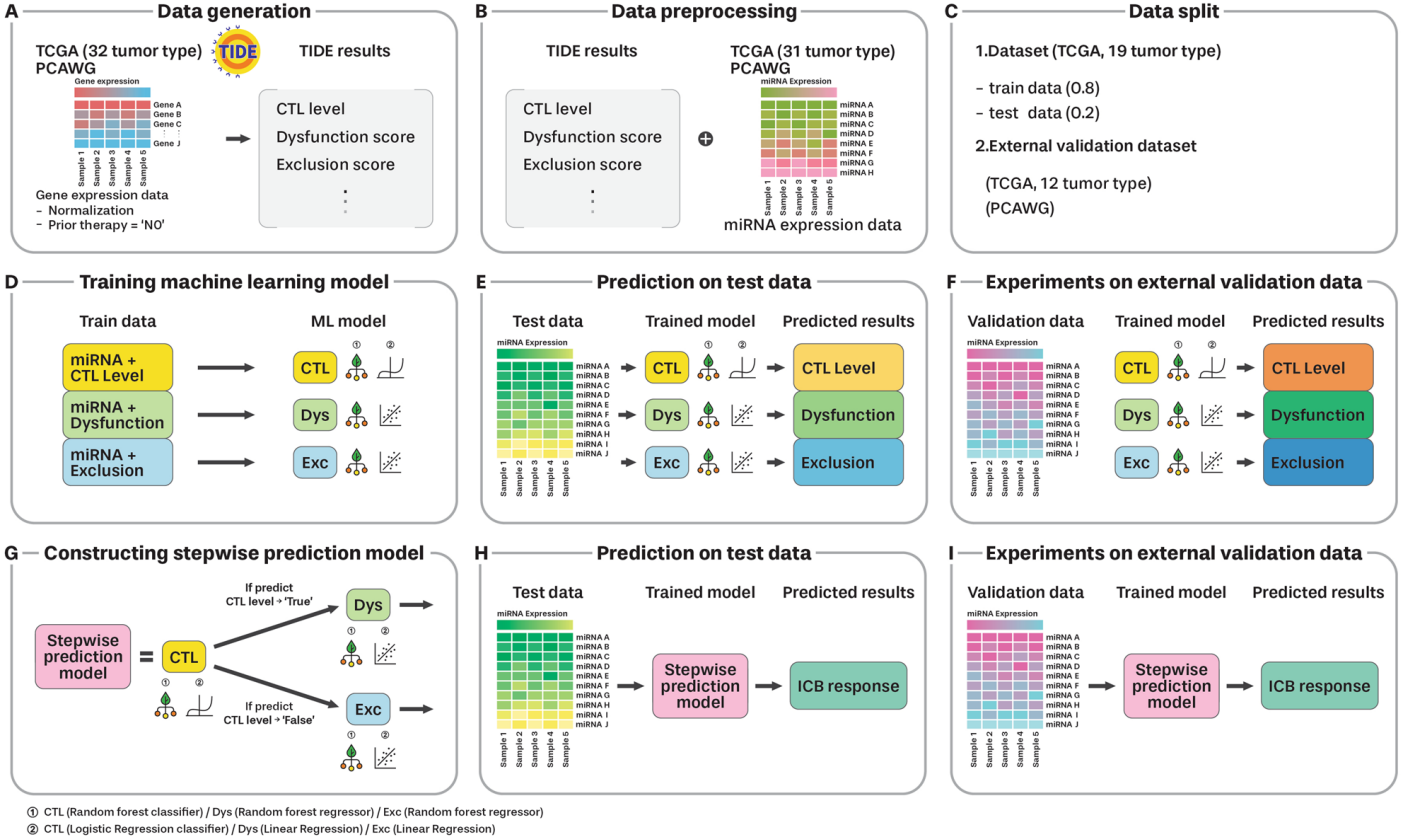
Workflow for ICB response prediction based on microRNA expression profiles. (**A**) Gene expression data is preprocessed by performing normalization and prior therapy screening. Subsequently, ICB response prediction results are obtained using TIDE method. (**B**) The TIDE results and miRNA expression data are aligned based on sample ID. (**C**) The data are divided into two groups; train/test and external validation. (**D**) The three machine learning models for predicting CTL levels, dysfunction scores, and exclusion scores, are trained. (**E**) Each trained model utilizes test data to obtain predicted values and measure its performance. (**F**) Each trained model uses external validation data to obtain predicted values and measure the performance. (**G**) The final ICB response is estimated based on the stepwise prediction by combining the trained models. (**H**) The final combined stepwise model uses test data to obtain the predicted result value and measure performance. (**I**) The external validation data is used for the final combined model to obtain the predicted result value and measure performance.

In this study, we developed a stepwise machine-learning model to predict ICB responses in patients with tumors based on miRNA expression profiles (Fig. 1). The model has two steps: the first step is to predict cytotoxic T lymphocyte (CTL) levels, and the second step is to predict T cell dysfunction and exclusion scores. Moreover, we employed SHapley Additive exPlanations (SHAP)^20,21^ for the interpretability of our machine learning model and analyzed the intrinsic mechanism guiding our predictions. Our study had the following objectives: first, to assess the efficacy of the ICB response prediction model using miRNA expression data, and second, to uncover the miRNAs that are closely related to ICB responses using SHAP analysis. Furthermore, we analyzed the biological functions of the genes targeted by these miRNAs and determined their potential impact on the immune systems of patients with tumors.

## Results

### ICB response prediction using miRNA expression profiles

**Figure 2:**
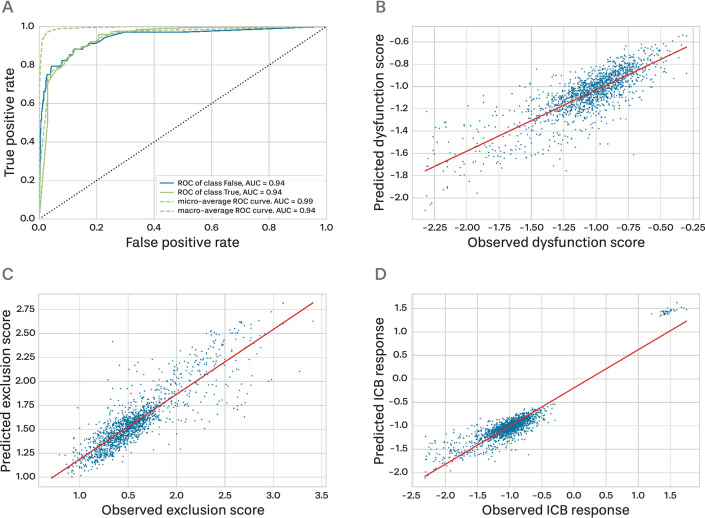
Predicted results for each model learned using miRNA expression profiles. (**A**) ROCAUC of the random forest classifier that predicts the CTL level. The class “True” signifies the high group and “False” signifies the low group. (**B**) Scatterplot of the random forest regression model for predicting the dysfunction score. The red line indicates the regression line. (**C**) Scatterplot of the random forest regression model for predicting the exclusion score. The red line indicates the regression line. (**D**) Scatterplot of the stepwise prediction model predicting ICB response based on the TIDE score. The red line indicates the regression line.

We compiled predictive ICB responses using the TIDE model and miRNA expression profiles from 7721 samples across 19 different tumor types within The Cancer Genome Atlas (TCGA) dataset (Table 1). To predict immunotherapy response using miRNA expression profiles, we first developed a random forest classifier to determine CTL levels. The optimal parameters for the random forest classifier were determined through a grid search with tenfold cross-validation (Table 2). Using the identified optimal parameters, we trained random forest classifiers on the designated training data and rigorously assessed the predictive performance on the independent test data. The results showed that the random forest classifier predicted the CTL levels well, with an AUC of 0.9400 (Fig. 2A). Furthermore, when evaluating the performance using the F1 score and Balanced AUC indicators, high performance was confirmed, with an F1 score of 0.9849 and a Balanced AUC of 0.7182.

**Table 1:** Abbreviation for tumor type and number of samples from The Cancer Genome Atlas (TCGA) gene and microRNA (miRNA) expression.

Tumor type	Abbreviation	Number of samples (gene expression)	Number of samples (miRNA expression)
Breast invasive carcinoma	BRCA	1217	1202
Kidney renal clear cell carcinoma	KIRC	607	592
Uterine corpus endometrial carcinoma	UCEC	583	575
Head and neck squamous cell carcinoma	HNSC	546	569
Lung adenocarcinoma	LUAD	585	564
Brain lower-grade glioma	LGG	529	530
Lung squamous cell carcinoma	LUSC	550	523
Ovarian serous cystadenocarcinoma	OV	379	498
Stomach adenocarcinoma	STAD	407	477
Colon adenocarcinoma	COAD	512	461
Skin cutaneous melanoma	SKCM	472	452
Bladder urothelial carcinoma	BLCA	430	432
Liver hepatocellular carcinoma	LIHC	424	425
Kidney renal papillary cell carcinoma	KIRP	321	326
Cervical squamous cell carcinoma and endocervical adenocarcinoma	CESC	309	312
Sarcoma	SARC	265	263
Pancreatic adenocarcinoma	PAAD	182	183
Esophageal carcinoma	ESCA	173	198
Uveal Melanoma	UVM	80	80
Glioblastoma multiforme*	GBM	173	5
Acute Myeloid Leukemia**	LAML	151	188

*GBM was not used in our experiments because the miRNA expression data were insufficient.

**LAML was not employed in our experiments because it was not a solid tumor.

**Table 2:** Parameters of each model tuned through GridSearchCV (tenfold cross validation).

Model	Best parameter
RFC* [CTL]** (All***)	(criterion = 'entropy', max_features = None, n_estimators = 250)
Logistic* [CTL] (SHAP 0.01***)	(class_weight: None, penalty: 'l2', solver: 'liblinear')
Logistic [CTL] (SHAP 0.02***)	(class_weight: None, penalty: 'l2', solver: 'liblinear')
RFR* [Dys]** (All)	(max_depth = 25, n_estimators = 700)
Linear* [Dys] (SHAP 0.01)	(default)
Linear [Dys] (SHAP 0.02)	(default)
RFR [Exc] (All)	(max_depth = 20, n_estimators = 300)
Linear [Exc] (SHAP 0.01)	(default)
Linear [Exc] (SHAP 0.02)	(default)
Stepwise model [CTL → Dys] (all)	RFC [CTL]: (criterion = 'entropy', max_features = None, n_estimators = 250) RFR [Dys]: (max_depth = 25, n_estimators = 700)
Stepwise model [CTL → Exc] (all)	RFC [CTL]: (criterion = 'entropy', max_features = None, n_estimators = 250) RFR [Exc]: (max_depth = 20, n_estimators = 300)
Stepwise model [CTL → Dys] (SHAP 0.01)	Logistic [CTL]: (class_weight: None, penalty: 'l2', solver: 'liblinear') Linear [Dys]: (default)
Stepwise model [CTL → Exc] (SHAP 0.01)	Logistic [CTL]: (class_weight: None, penalty: 'l2', solver: 'liblinear') Linear [Exc]: (default)
Stepwise model [CTL → Dys] (SHAP 0.02)	Logistic [CTL]: (class_weight: None, penalty: 'l2', solver: 'liblinear') Linear [Dys]: (default)
Stepwise model [CTL → Exc] (SHAP 0.02)	Logistic [CTL]: (class_weight: None, penalty: 'l2', solver: 'liblinear') Linear [Exc]: (default)

*RFC: Random forest classifier, RFR: Random forest regression model, Logistic: Logistic regression classifier, Linear: Linear regression model.

**[CTL]: model for predicting CTL level, [Dys]: model for predicting dysfunction score, [Exc]: model for predicting exclusion score, [CTL →  Dys]: model for predicting the ICB response using [Dys] model when [CTL] model predicts the CTL level as high, [CTL → Exc]: model for predicting the ICB response using [Exc] model when [CTL] model predicts the CTL level as low.

***All: All miRNA features, SHAP 0.01: miRNAs with feature importance of mean (|Shapley values|) > 0.01, SHAP 0.02: miRNAs with feature importance of mean (|Shapley values|) > 0.02.

Next, we predicted the dysfunction and exclusion scores based on random forest regression. A grid search with tenfold cross-validation was performed to determine the optimal parameters for random forest regression (Table 2). Employing the optimal parameters, two random forest regression models to predict the dysfunction and exclusion scores were independently learned from the training data. The predictive results with the independent test datasets showed that the MSE of the regression model for predicting the dysfunction and exclusion scores were both 0.0361. The Pearson correlation coefficient (PCC) between the observed and predicted values was also calculated. The PCC for the dysfunction score prediction model was 0.8158 and that for the exclusion model was 0.8704. This indicated a strong positive correlation between the predicted and actual values in both models (Fig. 2B,C).

Finally, we predicted the ICB responses based on the TIDE score by combining the two-step machine learning model, constructed a random forest classifier for CTL prediction, and random forest regression models for the dysfunction and exclusion scores. The MSE of the combined stepwise model was 0.0360. Furthermore, the PCC between the observed and predicted values exhibited a strong positive correlation of 0.9270 (Fig. 2D).

### Identification of miRNAs with high feature importance

**Figure 3:**
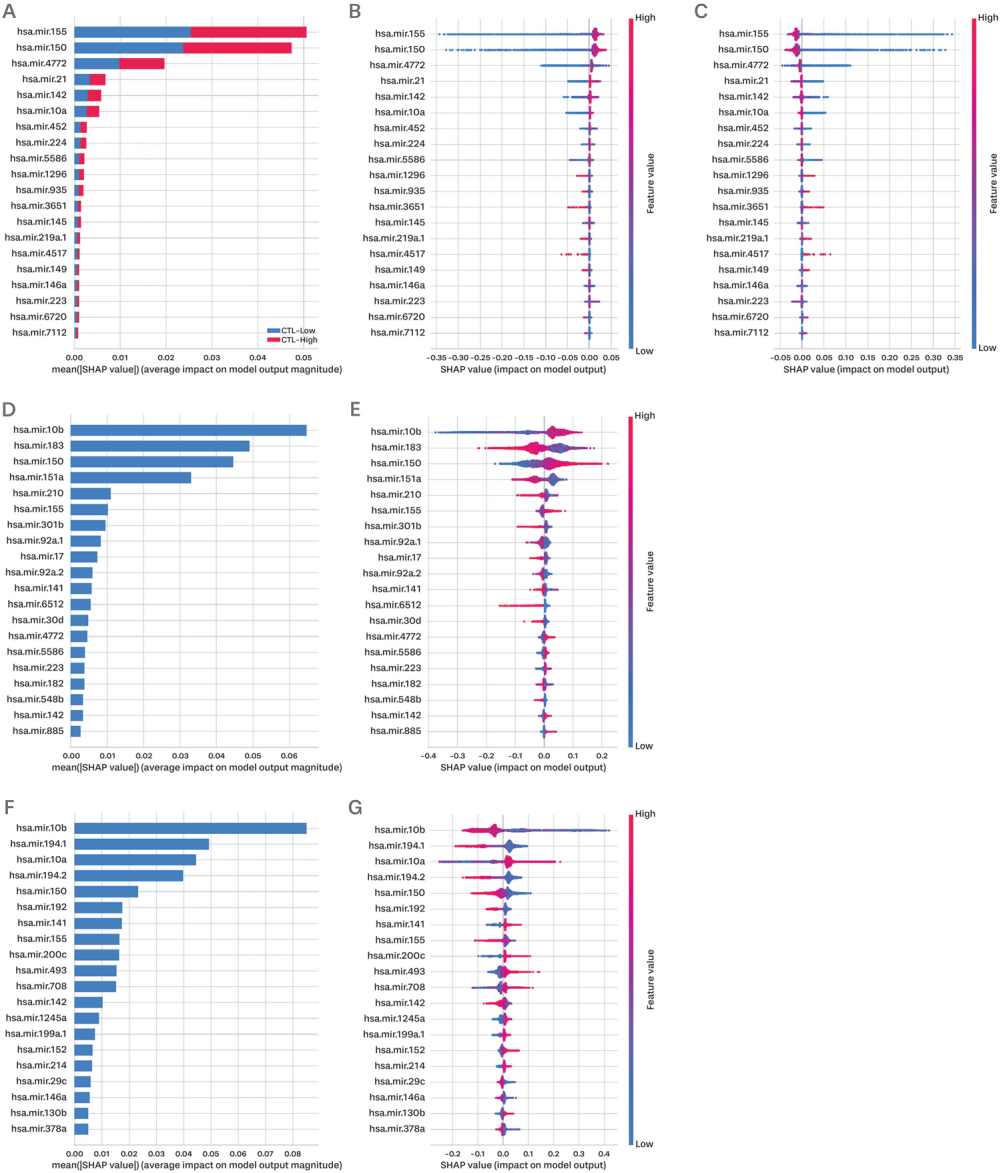
Shapley value plot for exhibiting feature importance. (**A**) SHAP feature importance for the random forest classifier to predict CTL level, (**B**) summary plot for the random forest classifier when the CTL prediction model predicts the CTL level is high, (**C**) summary plot for the random forest classifier when the CTL prediction model predicts the CTL level is low, (**D**) SHAP feature importance for random forest regression to predict dysfunction score, (**E**) summary plot for random forest regression to predict dysfunction score, (**F**) SHAP feature importance for random forest regression to predict exclusion score, and (**G**) summary plot for random forest regression to predict exclusion score. (**A**, **D**, **F**) are plots that arrange features based on the average of the absolute Shapley values, which serve as indicators of feature importance. (**B**, **C**, **E**, **G**) are summary plots that depict feature importance and feature effects simultaneously. Each point signifies the Shapley value of the feature and instance. The x-axis represents the Shapley value, and the y-axis represents each feature. The color of each point corresponds to the high and low feature values (i.e., miRNA expression values).

Thereafter, we used SHAP, an interpretable machine learning approach, to analyze the results of our machine learning models. Using SHAP analysis, we identified informative miRNAs that contributed to the prediction of target values. Figure 3 shows the top 20 miRNAs ranked according to their feature importance scores in each model.

For CTL-level prediction based on a random forest classifier, hsa-miR-155 was the most informative feature with the highest Shapley value. In particular, focusing on high and low CTL predictions, the expression of hsa-mir-155 was positively associated with CTL-level prediction (Fig. 3B,C). Notably, miR-155 is an essential factor orchestrating the CD8 + T cell response in cancer, and its overexpression has been associated with the enhancement of the anti-tumor response^22,23^. hsa-miR-150, which had the second-highest impact on model predictions, exhibited a similar trend. miR-150 also plays a crucial role in the differentiation and functional regulation of CD8 + T cells^24^. The absence of miR-150 leads to a decline in the killing ability of CD8 + T cells^24^. In addition, hsa-miR-4772, hsa-miR-21, hsa-miR-142, and hsa-miR-10a were also identified with notably high Shapley values.

In the random forest regression model used to predict the dysfunction score, the miRNA with the highest Shapley value was hsa-miR-10b. The Shapley value of hsa-mir-10b was negative when its expression was low, and positive when its expression was high (Fig. 3E). This indicated a positive correlation between hsa-miR-10b expression and dysfunction prediction. In contrast, hsa-miR-183 negatively correlated with dysfunction prediction. Both miR-150 and miR-155 showed positive correlations in dysfunction predictions and played an important role in dysfunction mechanisms, as well as in CTL level predictions. Furthermore, miR-151a and miR-210 exhibit negative correlations, similar to those of miR-183.

In the random forest regression model predicting the exclusion score, hsa-miR-10b also showed the largest Shapley value (Fig. 3G); however, it exhibited a negative correlation with hsa-miR-10b and the exclusion prediction, in contrast to the dysfunction prediction model. This observation serves as an example of how exclusion prediction, which has a mechanism opposite to that of dysfunction, is negatively correlated with dysfunction prediction. In contrast to the dysfunction results, hsa-miR-150 and hsa-miR-155 demonstrated opposite behaviors in exclusion prediction. Additionally, hsa-miR-10a, which was also identified in the CTL-level prediction, showed a positive correlation with exclusion prediction and played an important role in model prediction. Furthermore, the expression level of miR-194-1 and miR-194-2 is negatively correlated to the exclusion prediction.

### Prediction of ICB response using informative miRNAs

Next, we verified whether ICB response could be predicted using a small number of informative miRNAs. We selected miRNAs with an average absolute Shapley value of 0.01 or higher (SHAP 0.01). Using this criterion, three miRNAs were identified in the CTL model, five miRNAs in the dysfunction prediction model, and 12 in the exclusion prediction model (Fig. 3A,D,F). Because only a limited number of features were used to construct the models, we employed a simple algorithm to predict immunotherapy response.

To predict the CTL level, we applied logistic regression^25^ and determined the optimal parameters by conducting a grid search with tenfold cross-validation (Table 2). The model using the three informative miRNAs achieved an F1 score of 0.9805, a balanced accuracy of 0.7249, and an AUC value of 0.9300 (Fig. S1A). This analysis confirmed that a small subset of highly informative miRNAs displayed a similar performance in predicting CTL levels, even when a logistic regression model was utilized.

Subsequently, dysfunction and exclusion scores were predicted using a small number of informative miRNAs based on multiple linear regression. The obtained results showed that the MSE for the dysfunction model using the top miRNA (SHAP 0.01) was 0.0754 and that for the exclusion prediction model was 0.0840. The PCCs between the predicted and actual values were 0.5707 and 0.6638 for the dysfunction and exclusion prediction models, respectively (Figs. S1B,C). From these results, we confirmed that the performance was slightly degraded with a reduced number of features; however, the models still demonstrated comparable performance with only a small number of selected miRNAs.

Finally, to predict the ICB responses based on the TIDE scores, we applied a stepwise machine learning model by combining the logistic regression classifier for the CTL level and the linear regression model for dysfunction and exclusion scores. The MSE of the model that used the most informative miRNA (SHAP 0.01) was 0.0690. We also observed a strong positive correlation with informative miRNAs; the PCC of the top miRNA (SHAP 0.01) was 0.8457 (Fig. S1D).

Similarly, we applied robust criteria for the identification of informative miRNAs and verified whether having fewer miRNAs could result in the accurate prediction of immunotherapy response. We selected miRNAs with an average absolute Shapley value of 0.02 or higher (SHAP 0.02); two miRNAs were identified in the CTL model, four miRNAs in the dysfunction prediction model, and five miRNAs in the exclusion prediction model (Fig. 3A,D,F).

For CTL level prediction using logistic regression, the model obtained an F1 score of 0.9800, a balanced accuracy of 0.7459, and an AUC value of 0.91 (Fig. S2A). In addition, the models showed good performance for dysfunction and exclusion score prediction using linear regression. The MSE for dysfunction prediction was 0.0810 and that for exclusion prediction was 0.0984. The PCCs were 0.5220 and 0.5900 for the dysfunction score and exclusion score prediction models, respectively (Fig. S2B,C). Furthermore, for the ICB response prediction based on the TIDE scores using the two-step machine learning model combining logistic regression and linear regression, the MSE was 0.0753 and the PCC was 0.8595 (Fig. S2D). Although the performance was slightly lower than that of the model using all miRNAs for predicting the ICB response, these results suggest that informative miRNAs based on Shapley values still exhibit strong predictive capability, even with a limited number of miRNAs and relatively simple classification and regression models.

### Enrichment analysis for target genes of informative miRNAs

To examine the biological roles of the informative miRNAs, we predicted the target genes of the informative miRNAs selected by Shapley values using miRDB and TargetScan. A list of the genes targeted by the top miRNAs from each model is shown in Tables S1 and S2. We investigated the Kyoto Encyclopedia of Genes and Genomes (KEGG) pathways enriched in the target genes. Tables 3, 4, 5 and Tables S3–S5 show the results of enrichment analyses using the informative miRNAs (SHAP 0.01) of each model. The top 20 pathways are listed in Tables 3, 4, 5 in ascending order of *P*-values, and all KEGG pathways satisfying statistical significance (adjusted *P* value < 0.05) are shown in Tables S3–S5.

**Table 3:** Kyoto encyclopedia of genes and genomes (KEGG) pathways enriched by genes targeted by miRNAs with an average absolute Shapley value of 0.01 or higher in the random forest classifier predicting the CTL level (adjusted *P*-value < 0.05).

KEGG pathway	Adjusted *P*-value
TNF signaling pathway	0.0046
Hepatitis B	0.0063
IL-17 signaling pathway	0.0063
Fc epsilon RI signaling pathway	0.0067
Pathogenic *Escherichia coli* infection	0.0067
Adherens junction	0.0067
T cell receptor signaling pathway	0.0067
Pathways in cancer	0.0211
Osteoclast differentiation	0.0291
PI3K-Akt signaling pathway	0.0296
Lipid and atherosclerosis	0.0296
Cholinergic synapse	0.0296
Prostate cancer	0.0296
Fluid shear stress and atherosclerosis	0.0296
B cell receptor signaling pathway	0.0296
Growth hormone synthesis, secretion and action	0.0296

**Table 4:** Kyoto encyclopedia of genes and genomes (KEGG) pathways enriched by genes targeted by miRNAs with an average absolute Shapley value of 0.01 or higher in the random forest regression model predicting the dysfunction score (adjusted *P*-value < 0.05).

KEGG pathway	Adjusted *P*-value
Cushing syndrome	5.04E−06
Melanogenesis	5.04E−06
Wnt signaling pathway	9.80E−05
Axon guidance	9.80E−05
Dopaminergic synapse	1.25E−04
Adrenergic signaling in cardiomyocytes	1.68E−04
Long-term potentiation	2.57E−04
ErbB signaling pathway	5.63E−04
Cholinergic synapse	6.62E−04
Cortisol synthesis and secretion	7.59E−04
Glucagon signaling pathway	0.0012
Circadian entrainment	0.0016
Phospholipase D signaling pathway	0.0028
Growth hormone synthesis, secretion and action	0.0028
Neurotrophin signaling pathway	0.0028
Pathways in cancer	0.0043

**Table 5:** Kyoto encyclopedia of genes and genomes (KEGG) pathways enriched by genes targeted by miRNAs with an average absolute Shapley value of 0.01 or higher in the random forest regression model predicting the exclusion score (adjusted *P*-value < 0.05).

KEGG pathway	Adjusted *P*-value
Proteoglycans in cancer	7.02E−10
Axon guidance	1.53E−09
Pathways in cancer	8.50E−09
MAPK signaling pathway	6.93E−07
PI3K-Akt signaling pathway	7.77E−07
Rap1 signaling pathway	9.61E−07
Human cytomegalovirus infection	1.05E−06
Thyroid hormone signaling pathway	2.28E−06
Prostate cancer	6.95E−06
Endocytosis	1.16E−05
Neurotrophin signaling pathway	1.32E−05
Long-term potentiation	1.34E−05
Focal adhesion	1.79E−05
Renal cell carcinoma	1.93E−05
Cushing syndrome	1.93E−05
Cellular senescence	2.06E−05

The first-ranked KEGG pathway in the CTL-level prediction model was the TNF signaling pathway (Table 3). The following pathways are involved in the Hepatitis B and IL-17 signaling pathway. Hepatitis B is a significant contributor of hepatocellular carcinoma (HCC)^26^. Additionally, immune-related pathways such as the Fc epsilon RI signaling pathway and the T cell and B cell receptor signaling pathways were observed at the top. Enrichment analysis also revealed several other cancer-related terms, including “Pathways in cancer,” “PI3K-Akt signaling pathway,” “Prostate cancer,” “Renal cell carcinoma,” and “Pancreatic cancer” (Table S3). These results suggest a significant role for these miRNAs and their target genes in cancer and immunotherapy.

Tables 4 and S4 present the informative pathways identified using the dysfunction score prediction model. One of the most significantly enriched pathways was melanogenesis, which produces mutagenic intermediates that induce immunosuppression. The following term represents the Wnt signaling pathway and the ErbB signaling pathway. Moreover, our analysis identified various cancer-related terms, including “Hepatocellular carcinoma,” “Prostate cancer,” “Breast cancer,” and “Gastric cancer,” as well as “Pathways in cancer” (Table S4).

Tables 5 and S5 present the pathways identified using the exclusion score prediction model. The first pathway is “Proteoglycans in cancer,” which plays a significant role in regulating cytokine and chemokine expression on the cell surface. Moreover, various cancer-related pathways and terms, such as “MAPK signaling,” “PI3K-Akt signaling pathway,” and “Rap1 signaling pathway”, “Pathways in cancer,” “Prostate cancer,” “Renal cell carcinoma,” “Lung cancer,” and “Breast Cancer,” were also identified, along with immune-related pathways like “T cell and B cell receptor pathway” and “Helper T cell differentiation.” Furthermore, the presence of “PD-L1 expression” and the “PD-1 checkpoint pathway in cancer” indicate that the genes targeted by miRNAs are directly associated with immunotherapy.

Additionally, it was noted that several pathways related to the brain and neurons were observed, including “Axon guidance”^27^, a subfield of neurodevelopment associated with the process of neurons sending axons to reach accurate targets; “Neurotrophin signaling pathway”^28^, a protein that supports the survival, development, and function of neurons; “Long-term potentiation”^29^, a process that strengthens signal transmission between neurons; as well as “Dopaminergic synapse” and “Cholinergic synapse” (Table S5). This could be because the majority of CTL-low (exclusion) samples were involved in the TCGA LGG tumor type (Table S6).

Enrichment analysis results using the top miRNAs (SHAP value 0.02) of each model also identified diverse pathways related to cancer and immunity (Table S7-S9). These findings would provide valuable insights into the molecular mechanisms underlying exclusion and immune response regulation in cancer.

### Validation using other TCGA tumor types

We proceeded to validate the stepwise machine learning model based on a random forest trained on all miRNAs using data from 12 distinct tumor types not included in the previous training and test phases (Fig. 1F,I and Table S10). For the random forest classifier predicting CTL levels, we achieved an F1 score of 0.9912 and an AUC value of 0.9400 (Fig. S3A). When predicting the dysfunction and exclusion scores via random forest regression models, the MSE for the dysfunction score prediction model was 0.0478, and that for the exclusion score prediction model was 0.0641. The MSE value of the stepwise machine learning model for predicting the ICB response based on the TIDE score was 0.0475. Moreover, it could be observed that both the predicted value and the actual value showed a positive correlation (PCC = 0.8698). (Fig. S3B-D).

Furthermore, we validated the predictive potential of our immunotherapy response prediction model using small subsets comprising informative miRNAs (SHAP 0.01 and SHAP 0.02) by applying the same approaches to the 12 tumor types (Fig. 1F,I). The models employing informative miRNAs (SHAP 0.01) to predict CTL levels using logistic regression showed an F1 score of 0.9901 and an AUC of 0.9300 (Fig. S4A). In the dysfunction and exclusion score predictions using linear regression, the MSE were 0.0660 and 0.0677, respectively. Moreover, a positive correlation was observed between the predicted and actual values (PCC = 0.2899 and 0.4198, respectively) (Fig. S4B,C). Lastly, the stepwise model used to predict the ICB response based on the TIDE score with informative miRNA (SHAP 0.01) yielded an MSE of 0.0661 and a PCC of 0.8335 (Fig. S4D).

Additionally, the results of models with a smaller number of informative miRNAs and strict criteria (SHAP 0.02) revealed compelling outcomes. CTL-level prediction using the logistic regression classifier model showed an F1 score of 0.9904 and an AUC of 0.9300. (Fig. S5A). The linear regression models to predict the dysfunction and exclusion scores also achieved good performances, with the dysfunction score prediction model showing an MSE of 0.0585 and a PCC of 0.3822 and the exclusion score prediction model displaying an MSE of 0.0797 and a PCC of 0.2816 (Fig. S5B,C). In addition, for the prediction of the ICB response using the combined stepwise machine learning model with SHAP 0.02, the MSE was 0.0594 and the PCC was 0.8538 (Fig. S5D). Notably, the experimental results from the external validation datasets confirmed that not only did our model exhibit robust predictive performance regardless of tumor type, but the informative miRNAs were also useful for tumor immunotherapy response prediction.

### Validation using external independent dataset

We further validated the stepwise machine learning model trained on all miRNAs, using novel external independent data from PCAWG (Pancancer Analysis of Whole Genomes). The parameters of each model were set through grid search with tenfold cross-validation (Table S11). For the random forest classifier predicting CTL levels, we achieved an F1 score of 0.9589 and an AUC value of 0.9226 (Table S12). Regarding the prediction of dysfunction and exclusion scores through a random forest regression model, the MSE for the dysfunction score prediction model was 0.0245, and for the exclusion score prediction model, it was 0.0251 (Table S12). The MSE value of the stepwise machine learning model for predicting ICB response based on the TIDE score was 0.0248 (Table S12).

Furthermore, we identified informative miRNAs using the SHAP analysis in the PCAWG cohort (Fig. S6). In addition, we investigated which miRNAs were informative in each tumor type using SHAP (Table S13). It was noted that the informative miRNAs at TCGA cohorts were also similarly identified even at the PCAWG datasets, even though the direct comparison of the miRNAs is difficult because TCGA represents precursor miRNA expression and the PCAWG provides the mature forms. For instance, miR-150 demonstrated the significance in CTL and Dysfunction models. Furthermore, miR-155 was also assigned at a high ranking.

We also validated the predictability of ICB response prediction models in the PCAWG cohort using the informative miRNAs (SHAP 0.01 and SHAP 0.02) extracted from the TCGA cohort (Table S12). The model employing informative miRNAs (SHAP 0.01) achieved an F1 score of 0.9556 and an AUC of 0.9161 for predicting CTL levels via logistic regression. For dysfunction and exclusion score predictions using linear regression, the MSEs were 0.0371 and 0.0528, respectively. The stepwise model for predicting ICB response based on the TIDE score with informative miRNA (SHAP 0.01) yielded an MSE of 0.0376.

Similarly, the model utilizing informative miRNAs (SHAP 0.02) extracted from the TCGA cohort attained an F1 score of 0.9527 and an AUC of 0.9097 for predicting CTL levels via logistic regression. For dysfunction and exclusion score predictions using linear regression, the MSEs were 0.0364 and 0.0798, respectively. Finally, the stepwise model for predicting ICB response based on the TIDE score with informative miRNA (SHAP 0.02) yielded an MSE of 0.0364. The results with the external datasets from PCAWG further affirmed the effectiveness of the informative miRNAs in predicting ICB responses.

### Investigation in ICB responses based on each tumor type

Next, we employed the random forest-based ICB response prediction model on the TCGA cohort, stratified by tumor type, to investigate variations in the efficacy of ICI treatment in each tumor type. The parameters of each model were set through grid search with tenfold cross-validation (Table S11). The MSE values of the combined stepwise models for each tumor type ranged from 0.0093 to 0.0494 (Table S12). Notably, these results closely similar to the predictive performance derived from the entire tumor cohort. Thus, this suggests that the differences in ICI treatment response among various cancer types are minimal.

In addition, we investigated which miRNAs were informative in each tumor type using SHAP (Table S13). Even though there existed some differences in each tumor type, some informative miRNAs such as miR-150 and miR-155 were frequently observed at the highly-ranked miRNAs. This result indicates that these miRNAs are closely related to ICB responses across the tumor types.

Moreover, we also evaluated how well the stepwise model pre-trained using the whole 19 TCGA cohorts predicted the test data (20%) for each tumor type (Table S14). The MSE was ranged from 0.0113 to 0.1824 using total miRNAs. Using the information miRNAs (SHAP 0.01), the MSE was ranged from 0.0166 to 0.5530. Similarly, in the SHAP 0.02 model, the MSE was ranged from 0.0159 to 0.5562. These results showed the informative miRNAs were utilized for the prediction of ICB treatment responses even at a variety of cancer types.

## Discussion

In this study, we explored the feasibility of employing miRNAs as potential regulators of gene expression to predict clinical responses to ICB. To achieve this objective, we applied a supervised machine learning approach, specifically, a random forest model, and investigated informative miRNAs for ICB response prediction based on Shapley values.

In our experiment, we attempted to predict ICB response using the TIDE score as the target value. The TIDE score is an indicator for predicting the clinical response to ICB and was originally estimated from gene expression profiles. In our initial attempt, we attempted to predict the TIDE score directly using miRNA expression values; however, the experimental results were far from satisfactory, yielding an MSE of 0.2510. To overcome this problem, we devised a two-step approach that incorporates the CTL level and leverages either the dysfunction or exclusion scores. This strategic refinement led to a remarkable improvement in predictive performance, with an MSE of 0.0360 for predicting the TIDE score.

Additionally, by utilizing the SHAP approach to unravel the black-box issue in machine learning models, we elucidated the miRNAs that influenced model prediction. Through this process, we identified potential candidate miRNAs associated with tumor immunotherapy responses. Notably, miR-155 and miR-150 has emerged as prominent miRNAs. MiR-155 and miR-150 are pivotal as a regulatory factor essential for the CD8 + T cell response in cancer and its important role in the tumor microenvironment has been confirmed^22,23,30^.

Other top miRNAs for the CTL level prediction have been linked to tumors and immune responses. Decreased miR-4772 expression tends to increase the risk of recurrence and death from colon cancer^31^. miR-21 is considered one of the cancer-promoting 'oncomiRs' that target various tumor suppressor genes and its levels are indicative of immune cell activation^32^. In mice, miR-21 overexpression was shown to induce malignant B-cell lymphoma^32^. Furthermore, miR-142-3p, a member of the miR-142 family, is believed to be involved in the development and metastasis of various malignant tumors by targeting several mRNAs^33^.

Among the informative miRNAs for the dysfunction score prediction, miR-10b has been studied as a regulatory factor that causes cell movement and invasion when overexpressed in non-metastatic and metastatic breast tumor cells^34^. It has also been linked to immune escape through regulation of the immune microenvironment, leading to poor survival prognosis^35^. miR-183 reduces the expression of the tumor suppressor gene PTEN, contributing to its carcinogenic effects^36^. Furthermore, miR-194-1 and miR-194-2 can show a negative correlation with the tumor evasion mechanism of exclusion by reducing pancreatic tumor cell PD-L1 expression^37^.

The enrichment analyses on the target genes of the informative miRNAs further verified that the miRNAs would have potentials into the underlying the immunotherapy response in cancer. For instance, the top pathway was TNF signaling pathway in the CTL-level prediction model. TNF, also known as tumor necrosis factor, acts as both an inhibitor and a cytokine closely associated with cancer, playing a role in cancer cell growth, proliferation, invasion, and metastasis^38^. In particular, combining TNF blockade to increase the effectiveness of ICB has been explored as a novel treatment strategy. When this treatment strategy was applied to a mouse melanoma model, the prognosis was better than when using only ICB treatment^39^. Moreover, the IL-17 signaling pathway is also closely associated with tumor immune responses. IL-17 is a pro-inflammatory cytokine produced by CD4 + helper T cells and is strongly implicated in malignant tumor formation and metastasis^40^.

Melanogenesis, identified using the dysfunction score prediction model, produces mutagenic intermediates that induce immunosuppression and plays a crucial role in melanoma treatment by modulating the immune responses^41,42^. Aberrant Wnt signaling is closely related to various cancer types and influences tumor development by affecting the tumor microenvironment^43^. Furthermore, the ErbB signaling pathway plays a vital role in cancer development and progression, and targeting ErbB with tumor inhibitors is a widely used therapeutic approach^44^.

Proteoglycans in cancer, the first pathway in exclusion score prediction model plays a significant role in regulating cytokine and chemokine expression on the cell surface. It acts as a signaling coreceptor that influences the tumor microenvironment during the progression of solid and malignant tumors^45^. Additionally, it was noted that several pathways related to the brain and neurons were observed, including “Axon guidance”^27^, a subfield of neurodevelopment associated with the process of neurons sending axons to reach accurate targets; “Neurotrophin signaling pathway”^28^, a protein that supports the survival, development, and function of neurons; “Long-term potentiation”^29^, a process that strengthens signal transmission between neurons; as well as “Dopaminergic synapse” and “Cholinergic synapse” (Table S5). This could be because the majority of CTL-low (exclusion) samples were involved in the TCGA LGG tumor type (Table S6).

Furthermore, we showed that even a limited number of miRNAs could exhibit highly accurate predictions using independent datasets. The results across diverse cohorts highlighted the potentials of miRNA as a pivotal factor in predicting ICB responses.

Nonetheless, this study has some limitations. First, the TIDE scores used to predict the ICB responses were computationally estimated using the patients' gene expression profiles. In other words, the score may not accurately reflect the actual response to immunotherapy. Moreover, because the TIDE score was primarily designed to focus on gene expression data, the TIDE score might not reveal the effects of all other biological factors that might potentially affect tumor immunotherapy responses. However, the usefulness of the TIDE score for immunotherapy response prediction has been proven in previous studies^8^; thus, we used the TIDE score as the final target value in our investigation. In addition, in the search for machine learning model parameters, exhaustive verification of all possibilities of the model parameters is very time-consuming because of the nature of the continuous parameter values. Although rigorous efforts have been made through grid searches with cross-validation to identify optimized parameter values, it is still possible that some ideal combinations may not be explored. Moreover, at present, given the unavailability of real ICB-treated patients with high-throughput miRNA expression profiles, we should consider alternative indirect approaches to further validate our findings. Further validation using real patients with tumors treated with ICB would be necessary.

Finally, studies involving in vivo experiments investigating the impact of miRNAs can be crucial for confirming the clinical relevance and translational potential of our findings. Even though conducting such experiments is beyond the scope of this study, some previous works proved some potentials of the informative miRNAs by several wet-lab experiments. For example, in murine in vivo experiments, it was discovered that miR-142-5p modulates PD-L1 expression, suggesting that upregulation of miR-142-5p could potentiate the anti-tumor immune response^46^. Moreover, both murine and human NK cell experiments validated that therapeutic control of miR-150 enhances NK cell-mediated immunotherapy against cancer^47^. Furthermore, miR-155 has shown the efficacy in initiating an anti-tumor response within dendritic cell-based immunotherapy, resulting in a noteworthy enhancement in the survival rate of mice with colorectal cancer^48^.

Despite these limitations, our study demonstrates the potential utility of miRNAs as valuable predictors of immunotherapy response and suggests promising roles for informative miRNAs in tumor immunotherapy and microenvironments.

Looking ahead, future research directions would focus on refining predictive models through prospective validation studies, incorporating additional clinical variables, and exploring novel therapeutic targets identified through miRNA profiling. Moreover, efforts to elucidate the underlying mechanisms by which informative miRNAs regulate immune responses will deepen our understanding of tumor immunology. In addition, accessing larger datasets would enable the selection of precise miRNA biomarkers tailored to more specific groups such as tumor subtypes, sexes, age, and so on.

Furthermore, to the best of our knowledge, this study is the first computational model to predict the ICB responses. The current computational models predicting the ICB responses predominantly rely on genetic signatures, tumor mutation burden (TMB), and tumor PD-L1 levels assessed by immunohistochemistry (IHC)^4^. By integrating of some previously known predictive features and multi-omics datasets, including mRNA, DNA methylation, proteomic data, and miRNA, the prediction models of immunotherapy responses will be able to be enhanced. By addressing these challenges, it will be possible to advance personalized medicine in cancer treatment and improve patient outcomes in the era of immunotherapy.

## Materials and methods

### Data collection and preprocessing for gene expression profiles

To derive predictive immune response outcomes using the TIDE algorithm, bulk RNA-seq data were acquired from The Cancer Genome Atlas (TCGA) UCSC Xena browser (GDC repository) (https://gdc.xenahubs.net)^49^. A total of 21 tumor types in TCGA cohorts were gathered, and the same tumor types are available on the TIDE web browser (http://tide.dfci.harvard.edu/)^8^ (Table 1). Subsequently, gene expression data were used to predict the tumor immune response using the TIDE web browser. Gene expression values for all samples were normalized by subtracting the average log2 (FPKM + 1) value from each gene expression value^8^. Concurrently, ensemble ID for each gene was converted into gene symbol using the R package "org.Hs.eg.db" (version 3.16.0). Genes with duplicate symbols were replaced by calculating the average expression values.

Furthermore, a stringent filtering process was applied to the experiments, ensuring the inclusion of only TCGA samples lacking any prior treatment history, as it was not definitively confirmed whether immunotherapy had been administered to the samples. In addition, the study was limited to solid tumors; therefore, cases with acute myeloid leukemia were excluded. Finally, 8,037 samples harboring 35,096 genes across 20 tumor types were included.

### ICB response prediction based on TIDE

ICB response prediction was performed based on the TIDE method using TCGA gene expression data (Fig. 1A). These outcomes were conveyed through CTL level, dysfunction, exclusion values, and TIDE score. The CTL level was represented as either “True” or “False”, indicating high or low CTL levels, respectively. The TIDE scores were influenced by dysfunction and exclusion values. Specifically, when the CTL level was “True”, the dysfunction score was adopted as the TIDE score; conversely, if the CTL was “False”, the exclusion score was taken as the TIDE score. A sample with a positive TIDE score indicated that it was a non-responder, whereas a sample with a negative TIDE score was a responder.

### MicroRNA expression data

miRNA expression quantification (stem loop) data were also downloaded from the TCGA UCSC Xena browser (GDC repository) (https://gdc.xenahubs.net)^49^. This dataset comprised 1,881 miRNA expression values (log2 (RPM + 1)) per sample, encompassing 20 tumor types, identical to the TCGA gene expression data (Table 1). Normal samples were excluded from miRNA expression data. The GBM tumor type was excluded because the GBM included only five normal samples. In total, the dataset comprised 7721 samples from 19 tumor types (Fig. 1B).

For independent validation purposes, a validation dataset with 12 tumor types distinct from the 21 types available in the TIDE browser was used (Table S10). The validation dataset encompassed 1,947 samples (Fig. 1B,C).

### Developing ICB response prediction model

Random forest is an ensemble method that addresses overfitting by learning multiple decision trees^50^. This serves as a representative bagging model for both classification and regression tasks. In the classification model, the random forest predicts a class by selecting the most frequent class from among the predictions of multiple trees. For the regression model, the output was the average of the values obtained from individual trees as the prediction result.

A random forest classifier was employed to predict CTL levels based on miRNA expression values. For model learning and evaluation, the dataset comprising 7721 samples was divided into training (80%) and test (20%) datasets (Fig. 1C). The classifier model incorporates several parameters and its performance varies depending on the combination of these parameters. The training dataset was utilized to assess the performance of the model for each combination of the parameters through grid search (tenfold cross validation). Subsequently, the combination of parameters with the best performance was selected to the optimal parameters of the model. The parameters used are listed in Table 2. To assess the predictive performance of the trained model, F1 score and Balanced Accuracy were employed (Fig. 1D,E). The F1 score, a commonly used metric for data with unbalanced classes, represents the harmonic mean of Precision and Recall as follows:

$$F1 Score=2\times \frac{Recall\times Precision}{Recall+Precision}$$

The F1 score, which ranges from 0 to 1, combines the Precision and Recall values, both of which must be high to indicate good performance. Therefore, it is employed as the final performance evaluation indicator^51^.

Balanced accuracy is also one of the useful indicators when dealing with imbalanced datasets^52^, as follows:

$$Balanced \, Accuracy= \frac{Sensitivity+Specificity}{2}$$

Sensitivity represents the true-positive rate, indicating the model's ability to identify positive cases, and specificity denotes the true-negative rate, indicating the model's capacity to identify negative cases. Balanced Accuracy, ranging from 0 to 1, reflects the model's overall performance, with higher values indicating better performance.

To predict dysfunction and exclusion scores based on miRNA expression values, a random forest regression model was used. The regression model was fine-tuned through a grid search (tenfold cross validation) using the training dataset. The parameters ultimately utilized are listed in Table 2. The prediction performance of the regression models was assessed using MSE indicators (Fig. 1D,E). The MSE represents the average of the squared differences between the actual and predicted values. The formula is as follows:

$${\text{MSE}}= \frac{1}{N}\sum_{i=1}^{N}{({f}_{i}-{y}_{i})}^{2}$$

where *N* is the number of samples, *f*_*i*_ is the predicted value of sample *i*, and *y*_*i*_ is the actual value (target value) of sample *i*.

The ultimate computational goal of the study was to compute the ICB response, that is, the TIDE score, which depends on the CTL level and either the dysfunction or exclusion score. This process requires a stepwise combination of the predictive classifier for the CTL level and two regression models that predict the dysfunction and exclusion scores (Fig. 1G).

First, a random forest classifier was employed to predict the CTL. Subsequently, two distinct models come into play: random forest regression, which is responsible for predicting the dysfunction score, and the exclusion score. When the predicted CTL value was deemed true (indicating a high CTL level), the TIDE score was estimated using a random forest regression model to predict the dysfunction score. Conversely, if the predicted CTL value was assessed as false (corresponding to a low CTL level), the TIDE score prediction drew on the random forest regression model to predict the exclusion score. The predictive performance of the ICB response using the TIDE score was evaluated using MSE (Fig. 1H). All the implementations of the two-step machine learning models and performance evaluations were conducted using the Python scikit-learn library^53^.

### Feature importance of miRNAs in random forest models

The importance of miRNAs within the random forest models was assessed using SHapley Additive exPlanations (SHAP)^20,21^, an effective tool for elucidating the predictive outcomes of the black-box model, by leveraging Shapley values. Values were harnessed to measure the importance of the features (i.e., miRNAs) within each random-forest-based model employed in these studies. The Shapley value was calculated using the Python SHAP library (version 0.41.0).

For the informative miRNAs, the miRNAs exhibiting an average absolute Shapley value of 0.01 or higher were selected (SHAP 0.01). The selected informative miRNAs were investigated for their potential to accurately predict ICB responses using TIDE scores. Furthermore, the potentials were examined even at the strict criterion with a smaller number of informative miRNAs, with an average absolute Shapley value of 0.02 or higher (SHAP 0.02) (Fig. 1E,H).

When constructing a model utilizing informative miRNA (SHAP 0.01, SHAP 0.02), a simplistic approach was adopted due to the limited number of features relative to the number of samples, which mitigates overfitting risks. A logistic regression classifier model in charge of classification was used to predict CTL level prediction, and a linear regression model in charge of regression was used to predict Dysfunction and Exclusion scores.

For model training and evaluation, the dataset was identically divided into training (80%) and testing (20%) sets (Fig. 1C). The logistic regression classifier model for adjusting the CTL level prediction involved fine-tuning parameters such as class weight, penalty, and solver, through grid search (tenfold cross-validation) using the training set. As the linear regression model is a straightforward algorithm, no parameter adjustments were necessary. The approach to constructing a stepwise model for predicting ICB responses using a logistic regression classifier and a linear regression model mirrored the method employed in the previous random forest model. These models were also implemented and evaluated using the Python scikit-learn library^53^. The comprehensive list of parameters examined in all models is provided in Table S15 Moreover, the ultimate parameter configurations of the models for validation are detailed in Table 2 and Table S11. For reproducibility, detailed preprocessing steps and implementation information for all models are available on GitHub (https://github.com/dongyeon99/).

When constructing the machine learning models, the selected informative miRNAs and simpler methods were adopted: logistic regression for classification tasks and linear regression for regression tasks^25,53^. The miRNA expression value was normalized using scikit-learn StandardScaler package^53^ to apply these models.

### Target gene prediction and enrichment analysis

TargetScan^54^ and miRDB^55^ were used to identify genes with high feature importance. miRDB is an online database for miRNA target prediction, in which all predicted targets have a prediction score between 50 and 100. According to the authors' guidelines^55^, targets with a prediction score of 80 or higher were considered highly reliable. Thus, genes with a prediction score of 80 or higher were selected as miRNA targets by miRDB in our experiments. For concrete target prediction, the selected target genes were obtained by taking the intersection of the prediction results from miRDB and TargetScan. The enrichment analysis was performed using EnrichR (https://maayanlab.cloud/Enrichr/)^56^. Enrichment terms with an adjusted *P* value of less than 0.05 were finally selected.

### Additional independent validation data

For additional validation, bulk RNA-seq and mature miRNA expression data were acquired from PCAWG (Pan-Cancer Analysis of Whole Genomes) (https://pcawg.xenahubs.net)^49^. For RNA-seq, 1341 samples and 33831 genes were identified from the PCAWG dataset for inclusion in our analysis (Fig. 1A). By integrating the mature miRNA expression data, 775 samples were utilized in the validation experiment (Fig. 1B,C). Whereas the TCGA miRNA expression included precursor forms, the PCAWG miRNA data consisted of mature miRNAs. Thus, the prediction model was re-trained using the PCAWG mature miRNA expression data to investigate the informative miRNAs based on Shapley values. Furthermore, to test the validity of the trained models with informative precursor miRNAs (SHAP 0.01 and 0.02) from TCGA cohorts, its corresponding mature forms were all used.

### Supplementary Information

Supplementary Information.

## Data Availability

The miRNA expression data for the TCGA cohorts can be accessed at https://gdc.xenahubs.net. The ICB response results (TIDE) are available at http://tide.dfci.harvard.edu/. Additionally, the gene expression data for TCGA cohorts, which were used as input data for the TIDE method, can be found at https://gdc.xenahubs.net. The gene and miRNA data from PCAWG cohort can be accessed at https://pcawg.xenahubs.net. The ICB response values (TIDE scores) for each sample were obtained from the GitHub repository (https://github.com/dongyeon99/ML_immunotherapy_response).

## Code availability

The necessary codes and scripts for this analysis can be downloaded from https://github.com/dongyeon99/ML_immunotherapy_response.
