# Supplementary material for: Identifying microRNAs associated with tumor immunotherapy response using an interpretable machine learning model
Source: Sci Rep. 2024 Mar 14;14:6172. doi: 10.1038/s41598-024-56843-3 (PMC10940311; doi:10.1038/s41598-024-56843-3)
Supplement: Supplementary file 1 — Supplementary Information. [file 41598_2024_56843_MOESM1_ESM.zip › SupplementaryFiguresR1.pdf]

Identifying microRNAs associated with tumor immunotherapy response using an interpretable machine learning model

Dong-Yeon Nam[1] and Je-Keun Rhee[1 *]

[1]Department of Bioinformatics & Life Science, Soongsil University, Seoul, Republic of Korea

* Correspondence: jkrhee@ssu.ac.kr

Supplementary Materials

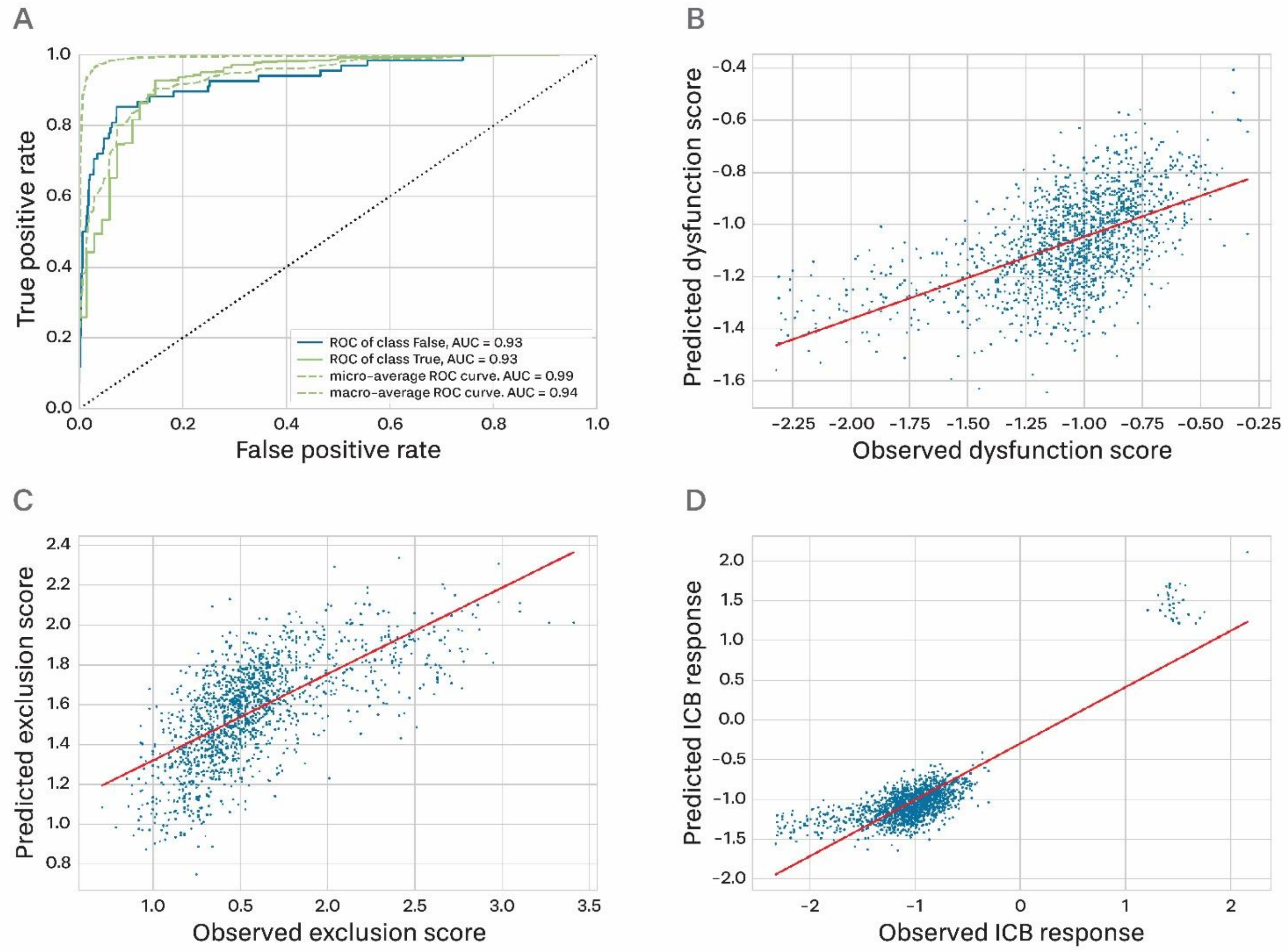

Figure S1. Prediction results of each model learned using miRNAs with an average value of 0.01 or higher of absolute Shapley value. (A) ROCAUC of the random forest classifier that predicts the CTL level. The class "true" signifies the high group, and "false" signifies the low group. (B) Scatterplot of the random forest regression model for predicting the dysfunction score. The red line indicates the regression line. (C) Scatterplot of the random forest regression model for predicting the exclusion score. The red line indicates the regression line. (D) Scatterplot of the stepwise prediction model predicting ICB response based on the TIDE score. The red line indicates the regression line.

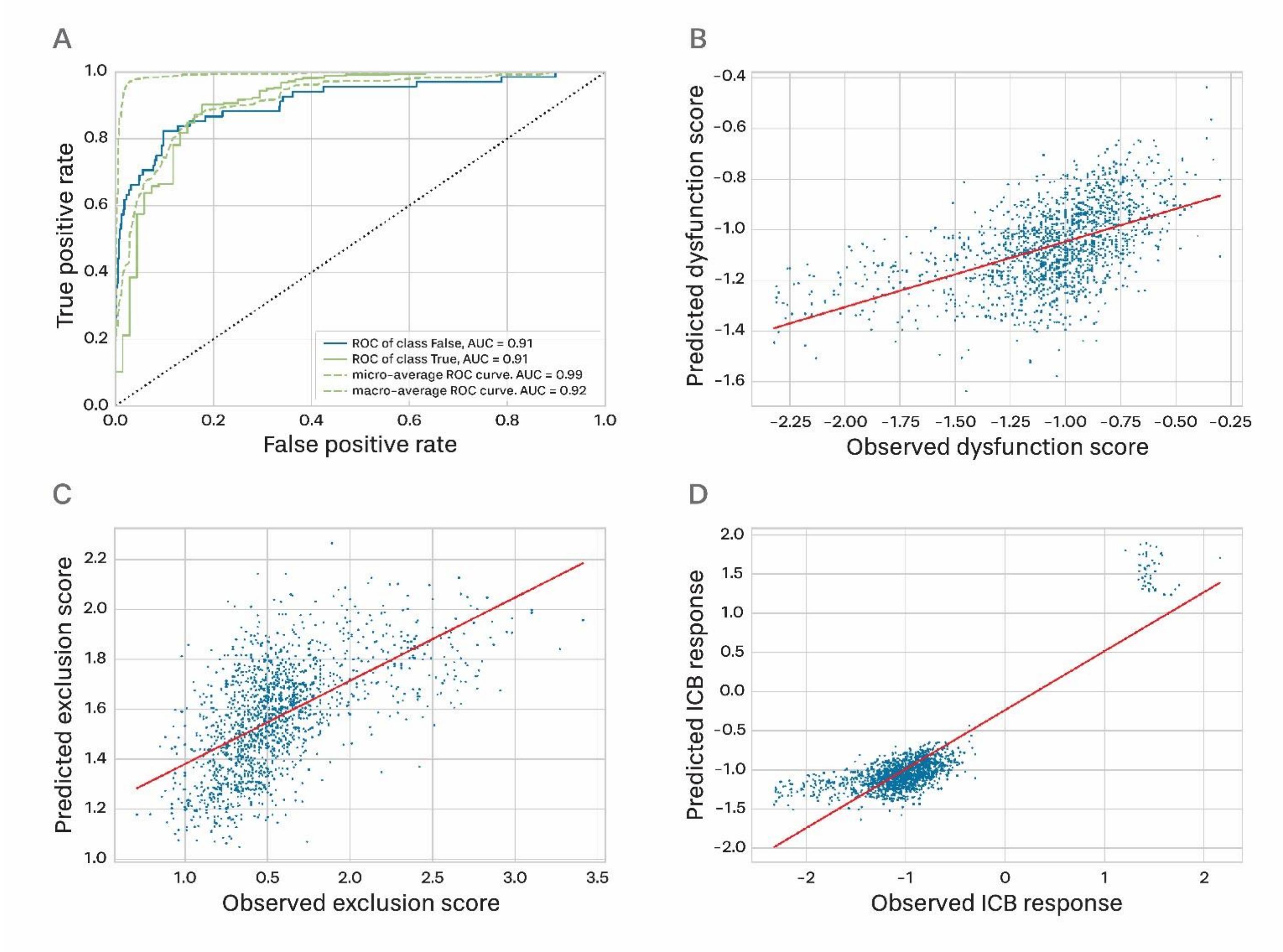

Figure S2. Prediction results of each model learned using miRNAs with an average value of 0.02 or higher of absolute SHAP value. (A) ROCAUC of the random forest classifier that predicts the CTL level. The class “true” signifies the high group and false signifies the low group. (B) Scatterplot of the random forest regression model for predicting the dysfunction score. The red line indicates the regression line. (C) Scatterplot of the random forest regression model for predicting exclusion score. The red line indicates the regression line. (D) Scatterplot of the stepwise prediction model predicting ICB response based on the TIDE score. The red line indicates the regression line.

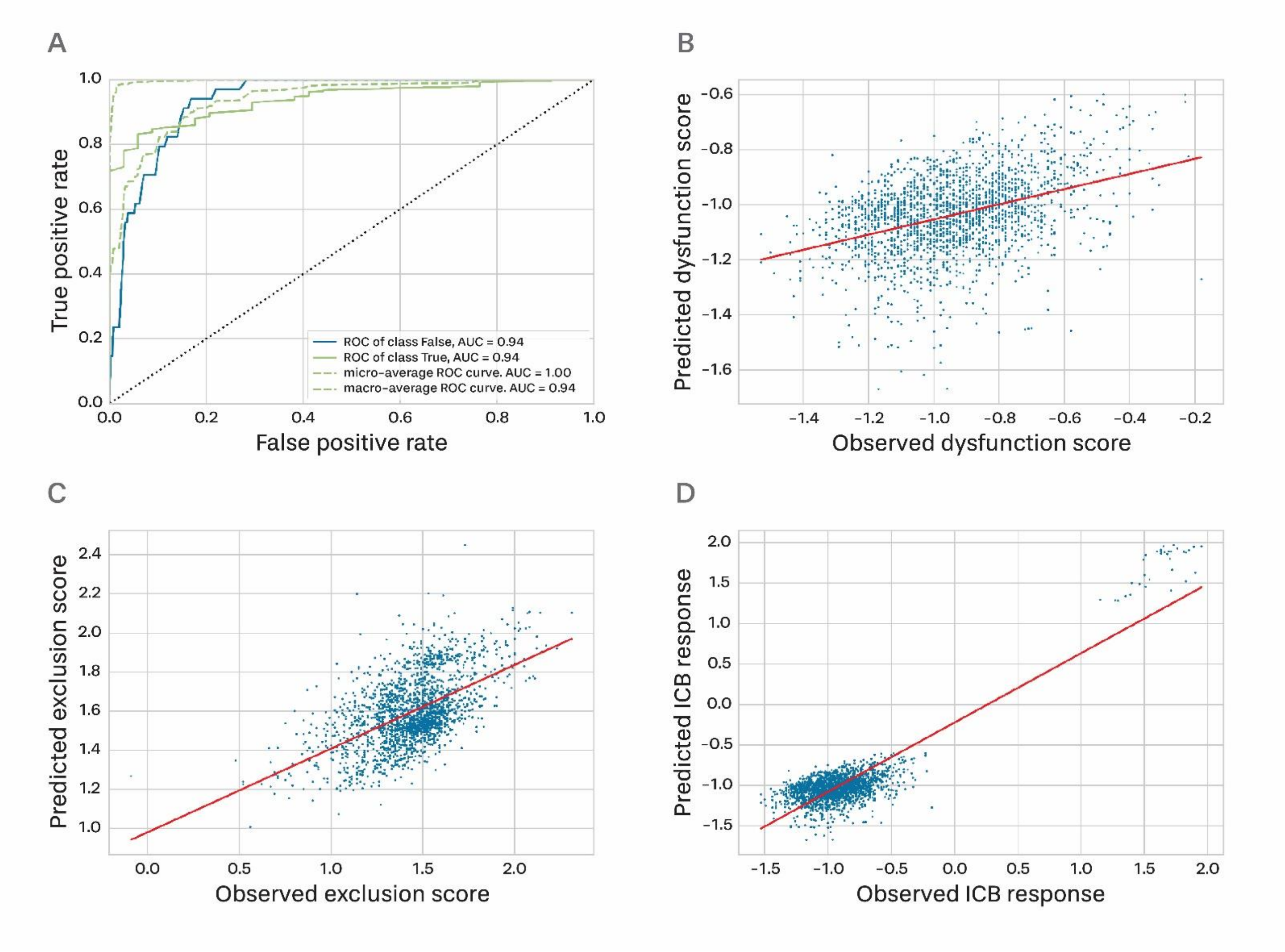

Figure S3. Prediction results of each model using external validation data (all miRNA). (A) ROCAUC of the random forest classifier that predicts the CTL level. The class “true” signifies the high group, and “false” signifies the low group. (B) Scatterplot of the random forest regression model for predicting the dysfunction score. The red line indicates the regression line. (C) Scatterplot of the random forest regression model for predicting the exclusion score. The red line indicates the regression line. (D) Scatterplot of the stepwise prediction model predicting ICB response based on the TIDE score. The red line indicates the regression line.

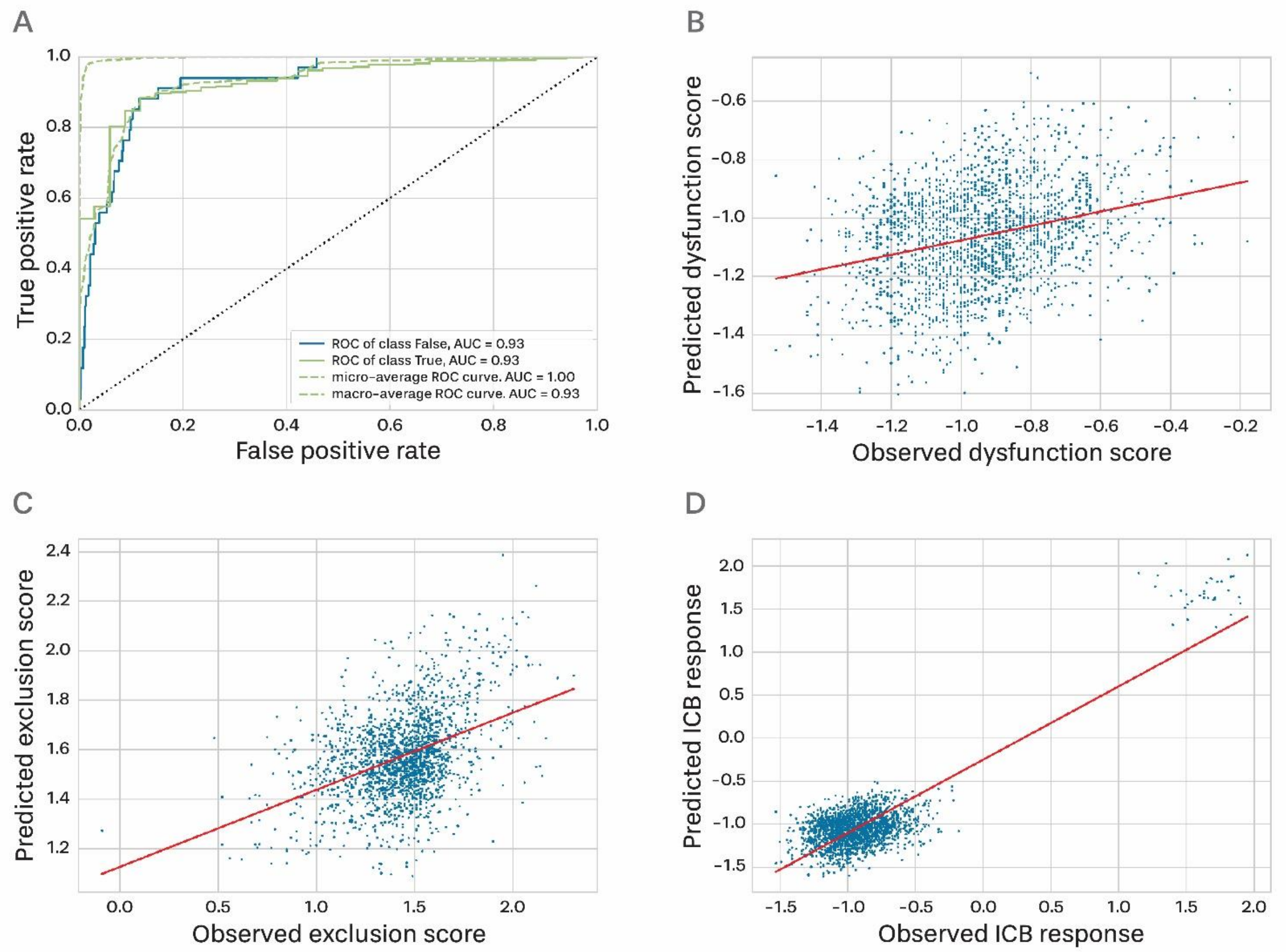

Figure S4. Prediction results of each model learned using external validation data (miRNA with an average absolute SHAP value of 0.01 or higher). (A) ROCAUC of the random forest classifier that predicts the CTL level. The class “true” signifies the high group and false signifies the low group. (B) Scatterplot of the random forest regression model for predicting the dysfunction score. The red line indicates the regression line. (C) Scatterplot of the random forest regression model for predicting the exclusion score. The red line indicates the regression line. (D) Scatterplot of the stepwise prediction model predicting ICB response based on the TIDE score. The red line indicates the regression line.

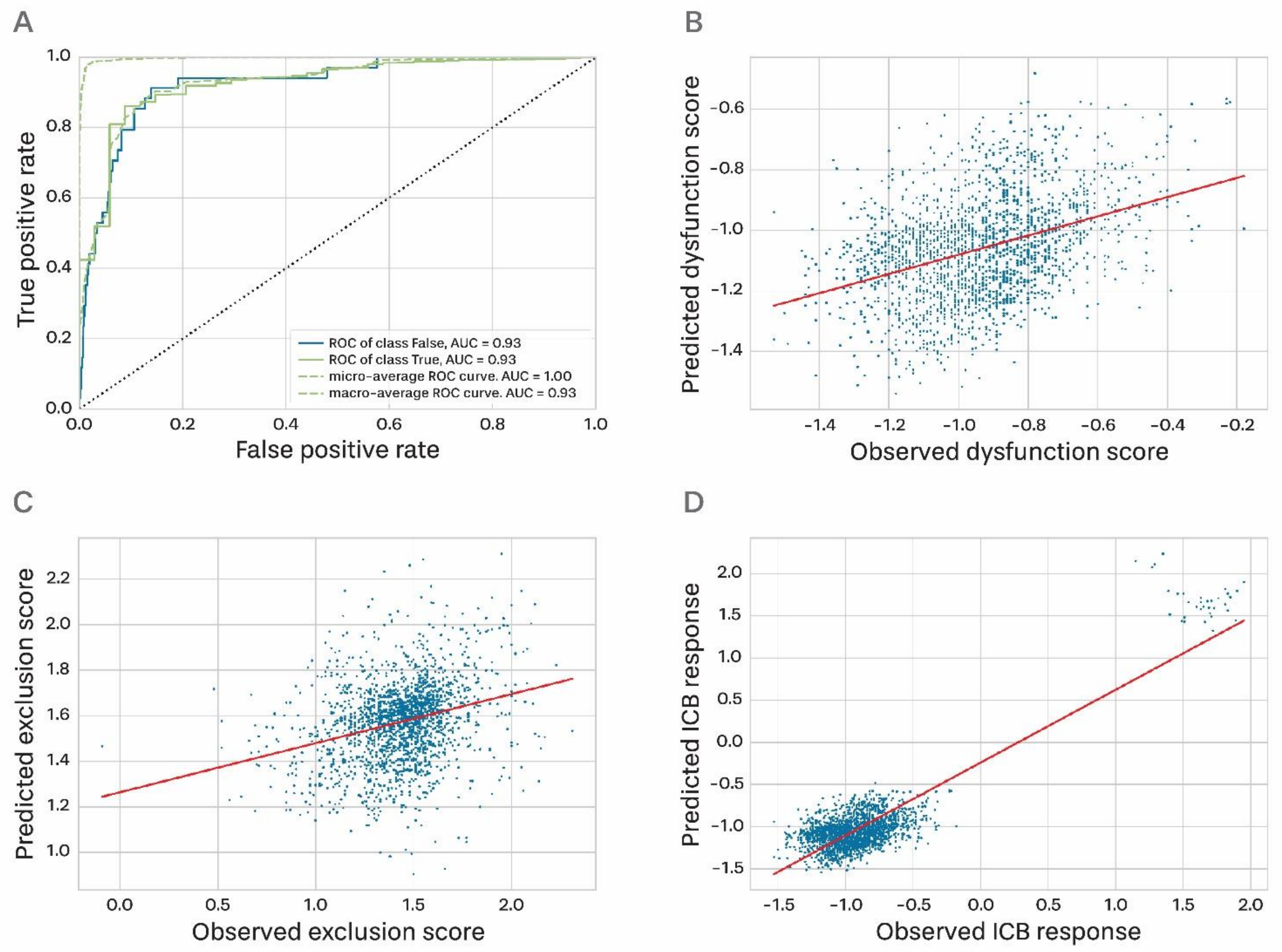

Figure S5. Prediction results of each model learned using external validation data (miRNA with an average absolute SHAP value of 0.02 or higher). (A) ROCAUC of the random forest classifier that predicts the CTL level. The class "true" signifies the high group and "false" signifies the low group. (B) Scatterplot of the random forest regression model for predicting the dysfunction score. The red line indicates the regression line. (C) Scatterplot of the random forest regression model for predicting the exclusion score. The red line indicates the regression line. (D) Scatterplot of the stepwise prediction model predicting ICB response based on the TIDE score. The red line indicates the regression line.

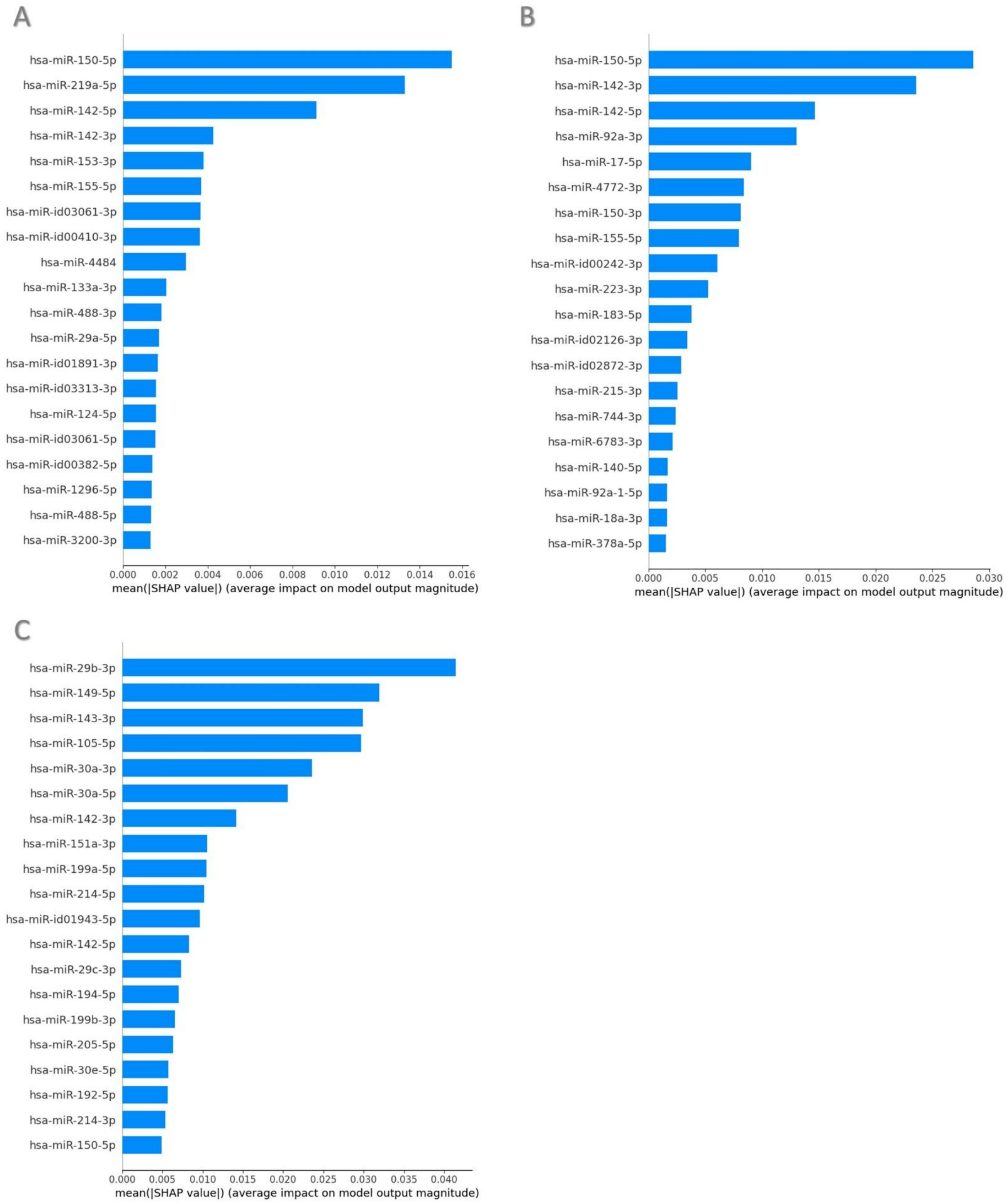

Figure S6. Shapley value plot for exhibiting feature importance for random forest models trained on PCAWG cohorts (A) SHAP feature importance for the random forest classifier to predict CTL level, (B) SHAP feature importance for random forest regression to predict dysfunction score, (C) SHAP feature importance for random forest regression to predict exclusion score.
